# *Moraxella catarrhalis* uses a twin-arginine translocation system to secrete the β-lactamase BRO-2

**DOI:** 10.1186/1471-2180-13-140

**Published:** 2013-06-19

**Authors:** Rachel Balder, Teresa L Shaffer, Eric R Lafontaine

**Affiliations:** 1 Department of Infectious Diseases, University of Georgia, Athens, GA 30602, USA; 2 Department of Microbiology, University of Georgia, Athens, GA 30602, USA

## Abstract

**Background:**

*Moraxella catarrhalis* is a human-specific gram-negative bacterium readily isolated from the respiratory tract of healthy individuals. The organism also causes significant health problems, including 15-20% of otitis media cases in children and ~10% of respiratory infections in adults with chronic obstructive pulmonary disease. The lack of an efficacious vaccine, the rapid emergence of antibiotic resistance in clinical isolates, and high carriage rates reported in children are cause for concern. Virtually all *Moraxella catarrhalis* isolates are resistant to β-lactam antibiotics, which are generally the first antibiotics prescribed to treat otitis media in children. The enzymes responsible for this resistance, BRO-1 and BRO-2, are lipoproteins and the mechanism by which they are secreted to the periplasm of *M. catarrhalis* cells has not been described.

**Results:**

Comparative genomic analyses identified *M. catarrhalis* gene products resembling the TatA, TatB, and TatC proteins of the well-characterized Twin Arginine Translocation (TAT) secretory apparatus. Mutations in the *M. catarrhalis tatA*, *tatB* and *tatC* genes revealed that the proteins are necessary for optimal growth and resistance to β-lactams. Site-directed mutagenesis was used to replace highly-conserved twin arginine residues in the predicted signal sequence of *M. catarrhalis* strain O35E BRO-2, which abolished resistance to the β-lactam antibiotic carbanecillin.

**Conclusions:**

*Moraxella catarrhalis* possesses a TAT secretory apparatus, which plays a key role in growth of the organism and is necessary for secretion of BRO-2 into the periplasm where the enzyme can protect the peptidoglycan cell wall from the antimicrobial activity of β-lactam antibiotics.

## Background

*Moraxella catarrhalis* is a Gram-negative bacterium primarily associated with otitis media in children and respiratory infections in adults with compromised lung function, particularly patients with Chronic Obstructive Pulmonary Disease (COPD). The organism is also readily isolated from the upper respiratory tract of healthy individuals and thus was considered a commensal bacterium until relatively recently. The rate of colonization by *M. catarrhalis* varies depending on many factors such as age, socioeconomic status, geography, and overall health condition. It has been reported that ~2/3 of children are colonized in their first year of life and 3-5% of adults carry the organism asymptomatically. Following initial colonization, there is a high rate of turnover, indicating continual clearance and re-colonization by new strains [1-27].

*Moraxella catarrhalis* possesses several virulence determinants that enable it to persist in the human respiratory tract. A number of molecules in the outer membrane have been shown to contribute to adherence, allowing *M. catarrhalis* to bind and colonize the host mucosa. These include LOS, UspA1, UspA2H, McaP, OMPCD, Hag/MID, MhaB1, MhaB2, MchA1, MchA2, and the type IV pilus [28-37]. In order to persist following colonization, *M. catarrhalis* possesses several mechanisms to evade the host immune system including resistance to complement. The best studied of these being UspA2 and UspA2H, which bind the C4-binding protein, C3 and vitronectin [38-41], as well as CopB, OMPCD, OmpE, and LOS [31,37,42,43].

*Moraxella catarrhalis* is often refractory to antibiotic treatment. Over 90% of isolates have been shown to possess a beta-lactamase, making them resistant to penicillin-based antibiotics [44-51], which are typically prescribed first to treat otitis media. The genes specifying this resistance appear to be of Gram-positive origin [52,53], suggesting that *M. catarrhalis* can readily acquire genes conferring resistance to additional antibiotics via horizontal transfer. Additionally, recent evidence has shown that *M. catarrhalis* persists as a biofilm *in vivo*, giving it further protection from antibiotic treatment and the host immune response [54-58].

The bacterial twin-arginine translocation (TAT) system mediates secretion of folded proteins across the cytoplasmic membrane. The TAT apparatus typically consists of three integral membrane proteins, namely TatA, TatB, and TatC. TatA forms the pore through which TAT substrates are secreted whereas TatB and TatC are important for binding and directing the substrates to the TatA pore. TatC acts as the gatekeeper for the secretion apparatus and specifically recognizes TAT substrates via a well-conserved signal sequence [59-62]. This signal sequence is similar to that of the general secretory system in that it possesses N- (neutral), H- (hydrophobic), and C- (charged) regions, but also contains a TAT-recognition motif (S/T-R-R-x-F-L-K) exhibiting nearly invariable twin arginines (RR) followed by two uncharged residues [59-62]. Proteins secreted via the TAT system are often, but not limited to, proteins that bind cofactors in the cytoplasm prior to transport, such as those involved in respiration and electron transport, and proteins that bind catalytic metal ions [59-62]. The TAT system has also been shown to secrete several factors important for bacterial pathogenesis including iron acquisition, flagella synthesis, toxins, phospholipases, and beta-lactamases [59,62-74]. In this study, we identified genes encoding a TAT system in *M. catarrhalis* and mutated these genes in order to elucidate the role of this translocase in the secretion of proteins that may be important for pathogenesis.

## Results and discussion

### Identification of *tatA, tatB* and *tatC* genes in *M. catarrhalis*

Analysis of the patented genomic sequence of *M. catarrhalis* strain ATCC43617 using NCBI’s tblastn service (http://blast.ncbi.nlm.nih.gov/Blast.cgi) identified an ORF (nucleotides 267,266 to 266,526 of GenBank accession number AX06766.1) that encodes a protein similar to the *tatC* gene product of *Pseudomonas stutzeri*[75] (expect value of 7e^-56^). TatC is the most highly-conserved component of the TAT system among organisms known (or predicted) to utilize this particular secretion apparatus [59-62]. TatC is located in the cytoplasmic membrane, typically contains 6 membrane-spanning regions, and plays a key role in recognizing the twin-arginine motif in the signal sequence of molecules secreted by the TAT system. The *M. catarrhalis* ATCC43617 *tatC*-like ORF specifies a 27-kDa protein of 247 amino acids, and analysis using the TMPred server (http://www.ch.embnet.org/software/TMPRED_form.html) revealed that it contains 6 potential membrane-spanning domains (data not shown).

**Figure 1:**
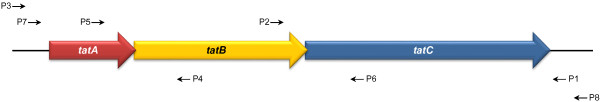
**Schematic representation of the *M. catarrhalis tatABC *locus.** The relative position of *tat*-specific oligonucleotide primers (P1-P8) used throughout the study is shown (see Methods section for details).

Sequence analysis upstream of the *M. catarrhalis tatC* ortholog identified gene products similar to other conserved components of the TAT system, TatA and TatB (Figure 1). The ORF immediately upstream encodes a 178-residue protein with a molecular weight of 20-kDa that resembles TatB of *Providencia stuartii *[76] (expect value of 3e^-8^). Upstream of the *M. catarrhalis tatB*-like gene, we identified an ORF specifying a 9-kDa protein of 77 aa that is most similar to TatA of *Xanthomonas oryzae *[77] (expect value of 2e^-5^). TatA and TatB are cytoplasmic proteins anchored to the cytoplasmic membrane via hydrophobic N-termini. TatB forms a complex with TatC often referred to as the twin-arginine motif recognition module, while TatA oligomerizes and forms a channel that is used to secrete TAT substrates [59-62]. Both *M. catarrhalis* ATCC43617 TatA (aa 4–21) and TatB (aa 5–21) orthologs are predicted to contain hydrophobic membrane-spanning domains in their N-termini using TMPred (data not shown). Together, these observations suggest that *M. catarrhalis* possesses a functional TAT system.

To assess the presence and conservation of the *tat* genes in other *M. catarrhalis* isolates, we amplified and sequenced these genes from strains O35E, O12E, McGHS1, V1171, and TTA37. The encoded gene products were then compared using ClustalW (http://www.ebi.ac.uk/Tools/msa/clustalw2/). Of note, the annotated genomic sequence of the *M. catarrhalis* isolate BBH18 has been published [78] and the predicted aa sequence of the TatA (MCR_0127, GenBank accession number ADG60399.1), TatB (MCR_0126, GenBank accession number ADG60398.1) and TatC (MCR_0125, GenBank accession number ADG60397.1) proteins were included in our comparative analyses. Overall, the TatA and TatC proteins are perfectly conserved. The TatB proteins divide the strains into two groups where O35E, McGHS1, TTA37, ATCC43617, and BBH18 are 100% identical to each other, while O12E and V1171 both contain the same aa substitution at residue 38 (S in lieu of G). We also noted that in all isolates examined, the *tatA* and *tatB* ORFs overlap by one nucleotide. A similar one-nucleotide overlap is also observed for the *tatB* and *tatC* coding regions. This observation suggests that the *M. catarrhalis tatA, tatB,* and *tatC* genes are transcriptionally and translationally linked.

### The *M. catarrhalis tatA*, *tatB* and *tatC* genes are necessary for optimal growth

To study the functional properties of the Tat proteins in *M. catarrhalis*, we constructed a panel of isogenic mutant strains of isolate O35E in which the *tatA, tatB* and *tatC* genes were disrupted with a kanamycin resistance (kan^R^) marker. Each mutant was also complemented with a plasmid encoding a wild-type (WT) copy of the mutated *tat* gene and/or with a plasmid specifying the entire *tatABC* locus. A growth defect was immediately noted in the *tat* mutants as ~40-hr of growth at 37°C was necessary for isolated colonies of appreciable size to develop on agar plates, compared to ~20-hr for the parent strain O35E. Hence, we compared the growth of the *tat* mutants to that of the WT isolate O35E in liquid medium under aerobic conditions. This was accomplished by measuring the optical density (OD) of cultures over a 7-hr period. In some of these experiments, we also plated aliquots of the cultures to enumerate colony forming units (CFU) as a measure of bacterial viability.

**Figure 2:**
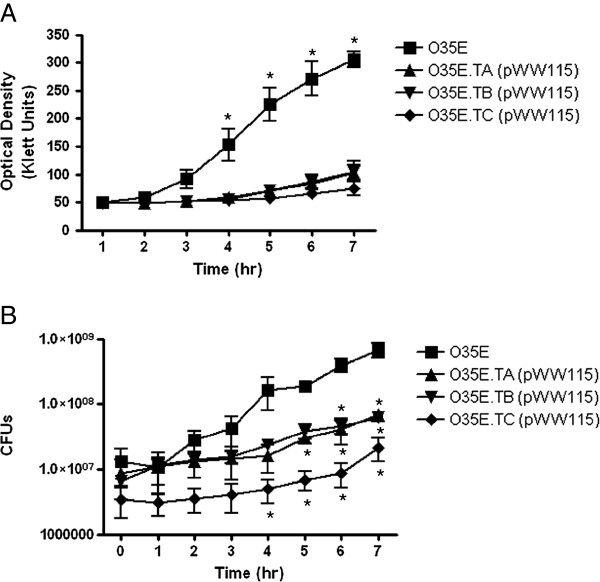
**Growth of the *M. catarrhalis *WT isolate O35E and *tat *mutant strains in liquid medium.** Plate-grown bacteria were used to inoculate sidearm flasks containing 20-mL of broth to an optical density (OD) of ~50 Klett units. The cultures were then incubated with shaking at a temperature of 37°C for seven hours. The OD of each culture was determined every 60-min using a Klett Colorimeter. Results are expressed as the mean OD ± standard error (**Panel A**). Aliquots (1-mL) were taken out of each culture after recording the OD, diluted, and spread onto agar plates to determine the number of viable colony forming units (CFU). Results are expressed as the mean CFU ± standard error (**Panel B**). Growth of the wild-type (WT) isolate O35E is compared to that of its *tatA* (O35E.TA), *tatB* (O35E.TB), and *tatC* (O35E.TC) isogenic mutant strains carrying the control plasmid pWW115. Asterisks indicate a statistically significant difference in the growth rates of mutant strains compared to that of the WT isolate O35E.

**Figure 3:**
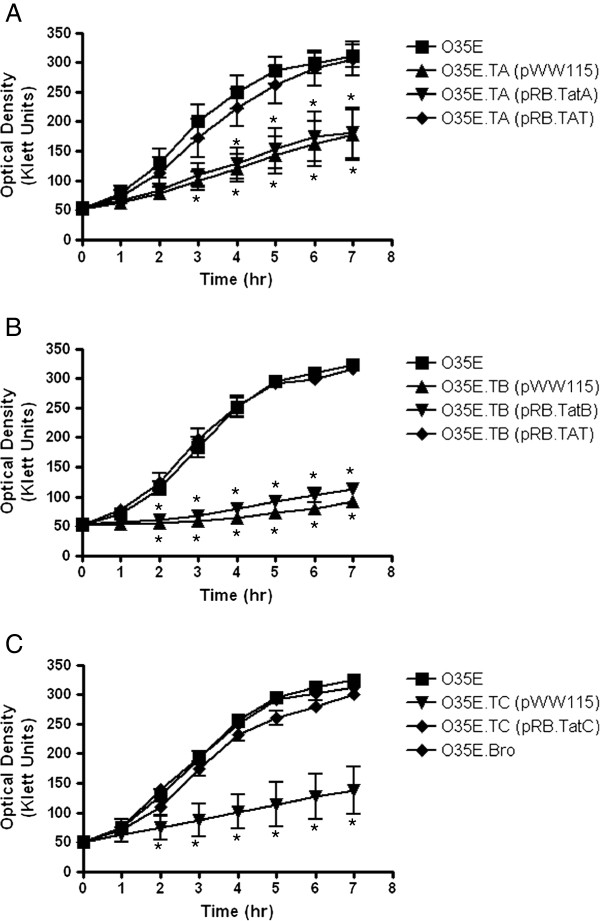
**Growth of the *M. catarrhalis *WT isolate O35E and *tat *mutant strains in liquid medium.** Plate-grown bacteria were used to inoculate sidearm flasks containing 20-mL of broth to an OD of 50 Klett units. The cultures were then incubated with shaking at a temperature of 37°C for seven hours. The OD of each culture was determined every 60-min using a Klett Colorimeter. **Panel A**: Growth of O35E is compared to that of its *tatA* isogenic mutant strain, O35E.TA, carrying the plasmid pWW115 (control), pRB.TatA (specifies a WT copy of *tatA*), and pRB.TAT (harbors the entire *tatABC* locus). **Panel B**: Growth of O35E is compared to that of its *tatB* isogenic mutant strain, O35E.TB, carrying the plasmid pWW115, pRB.TatB (specifies a WT copy of *tatB*), and pRB.TAT. **Panel C**: Growth of O35E is compared to that of its *tatC* isogenic mutant strain, O35E.TC, carrying the plasmid pWW115 and pRB.TatC (contains a WT copy of *tatC*). Growth of the *bro-2* isogenic mutant strain O35E.Bro is also shown. Results are expressed as the mean OD ± standard error. Asterisks indicate a statistically significant difference in the growth rates of mutant strains compared to that of the WT isolate O35E.

As shown in Figure 2A, the *tatA*, *tatB* and *tatC* mutants carrying the control plasmid pWW115 had lower OD readings than their progenitor strain O35E throughout the entire course of the experiments. Significant differences in the number of CFU were also observed between mutants and WT strains (Figure 2B). Together, these results demonstrate that mutations in the *tatA*, *tatB* and *tatC* genes substantially reduce the growth rate of *M. catarrhalis* cells. Complementation of the *tatA* (Figure 3A) and *tatB* (Figure 3B) mutants with plasmids encoding WT *tatA* (*i.e.* pRB.TatA) or *tatB* (*i.e.* pRB.TatB) did not rescue the growth phenotype of these strains. However, the construct pRB.TAT, which specifies the entire *tatABC* locus, restored growth of the *tatA* and *tatB* mutants to WT levels (Figure 3A and B). These results support the hypothesis that the *tatA, tatB* and *tatC* genes are transcriptionally and translationally linked due to the one nucleotide overlaps between the *tatA* and *tatB*, as well as the *tatB* and *tatC* ORFs. For the *tatC* mutant, O35E.TC, introduction of the plasmid pRB.TatC, which encodes only the *tatC* gene, is sufficient to restore growth to WT levels (Figure 3C). This finding is consistent with the above observations since *tatC* is located downstream of *tatA* and *tatB* (Figure 1), thus it is unlikely that a mutation in *tatC* would affect the expression of either the *tatA* or *tatB* gene product. A *tatC* mutation was also engineered in the *M. catarrhalis* isolate O12E. The resulting strain, O12E.TC, exhibited a growth defect comparable to that of the *tatC* mutant of strain O35E, and this growth defect was rescued by the plasmid pRB.TatC (data not shown). These results demonstrate that the importance of the TAT system to *M. catarrhalis* growth is not a strain-specific occurrence. Of note, all *tat* mutants carrying the control plasmid pWW115 grew at rates comparable to the mutants containing no plasmid (data not shown).

### The *tatA*, *tatB* and *tatC* genes are necessary for the secretion of β-lactamase by *M. catarrhalis*

**Figure 4:**
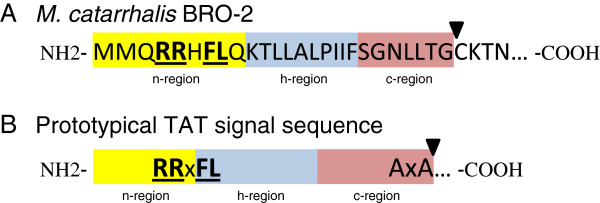
**Features of the *M. catarrhalis *BRO-2 signal sequence.** The *M. catarrhalis* ATCC43617 *bro-2* gene product was analyzed using the SignalP 4.0 server. **Panel A**: The first 30 amino acid of BRO-2 are shown. Residues 1–26 specify characteristics of a prokaryotic signal sequence, specifically neutral (n, highlighted in yellow), hydrophobic (h, highlighted in blue) and charged (c, highlighted in red) regions. The highly-conserved twin-arginine recognition motif is bolded and underlined. **Panel B**: Features of a typical TAT signal sequence where x represents any amino acid (adapted from [59]). The arrowheads indicate signal peptidase cleavage sites.

TAT-deficient mutants of *E. coli *[79] and mycobacteria [72-74,80] have been previously shown to be hypersensitive to antibiotics, including β-lactams. Moreover, the β-lactamases of *M. smegmatis* (BlaS) and *M. tuberculosis* (BlaC) have been shown to possess a twin-arginine motif in their signal sequences and to be secreted by a TAT system [74]. More than 90% of *M. catarrhalis* isolates are resistant to β-lactam antibiotics [44-51]. The genes responsible for this resistance, *bro-1* and *bro-2*, specify lipoproteins of 33-kDa that are secreted into the periplasm of *M. catarrhalis* where they associate with the inner leaflet of the outer membrane [52,53]. Analysis of the patented genomic sequence of *M. catarrhalis* strain ATCC43617 with NCBI’s tblastn identified the *bro-2* gene product (nucleotides 8,754 to 7,813 of GenBank accession number AX067438.1), which is predicted to encode a protein of 314 residues with a predicted MW of 35-kDa. The first 26 residues of the predicted protein were found to specify characteristics of a signal sequence (*i.e.* n-, h-, and c-region; see Figure 4A). Analysis with the LipoP server (http://www.cbs.dtu.dk/services/LipoP/) indicated a signal sequence cleavage site between residues 26 and 26 (*i.e.* TG^26▼^C^27^K) of BRO-2 (arrowhead in Figure 4A), which would provide a free cysteine residue for lipid modification of this lipidated β-lactamase [52]. Of significance, the putative signal sequence of BRO-2 contains the highly-conserved twin-arginine recognition motif RRxFL (Figure 4), thus suggesting that the gene product is secreted via a TAT system. Of note, analysis of *M. catarrhalis* BRO-1 sequences available through the NCBI database indicates that the molecules also contain the twin-arginine recognition motif (data not shown).

**Figure 5:**
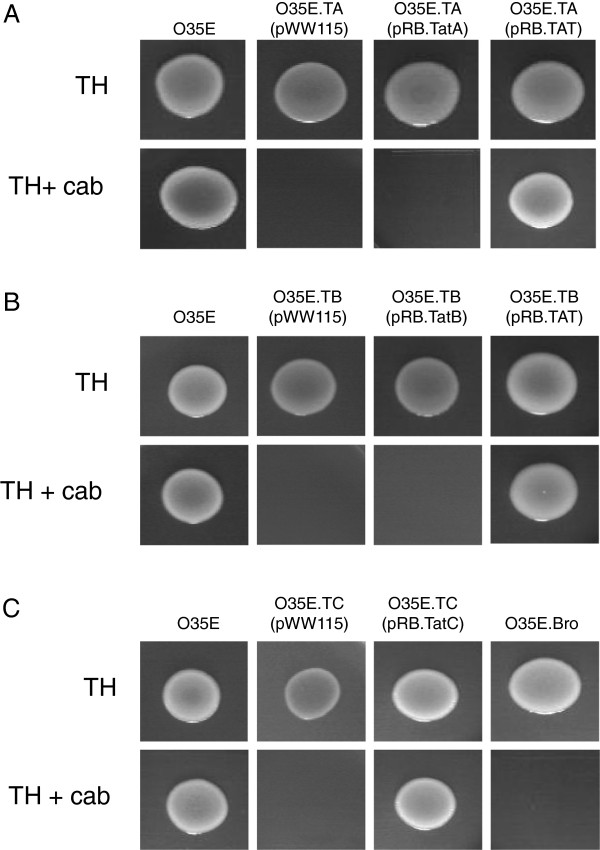
**Growth of the *M. catarrhalis *WT isolate O35E and *tat *mutant strains in the presence of the β-lactam antibiotic carbenicillin.** The ability of *tat* mutants to grow in the presence of carbenicillin (cab) was tested by spotting equivalent numbers of bacteria onto Todd-Hewitt agar plates supplemented with the antibiotic (TH + cab). As control, bacteria were also spotted onto agar plates without carbenicillin (TH). These plates were incubated for 48 hrs at 37°C to accommodate the slower growth rate of the *tat* mutants. **Panel A**: Growth of O35E is compared to that of its *tatA* isogenic mutant strain, O35E.TA, carrying the plasmid pWW115 (control), pRB.TatA (specifies a WT copy of *tatA*), and pRB.TAT (harbors the entire *tatABC* locus). **Panel B**: Growth of O35E is compared to that of its *tatB* isogenic mutant strain, O35E.TB, carrying the plasmid pWW115, pRB.TatB (specifies a WT copy of *tatB*), and pRB.TAT. **Panel C**: Growth of O35E is compared to that of its *tatC* isogenic mutant strain, O35E.TC, carrying the plasmid pWW115 and pRB.TatC (contains a WT copy of *tatC*). Growth of the *bro-2* isogenic mutant strain O35E.Bro is also shown. The results are shown as a composite image representative of individual experiments that were performed in duplicate on at least 3 separate occasions.

Based on these findings, we compared the ability of our panel of WT and *tat* mutant strains to grow in the presence of the β-lactam antibiotic carbenicillin. This was accomplished by spotting equivalent numbers of bacteria onto agar plates supplemented with the antibiotic. For comparison, bacteria were also spotted onto agar plates without carbenicillin. These plates were incubated for 48-hr at 37°C to accommodate the slower growth rate of *tat* mutants. In contrast to WT *M. catarrhalis* O35E, which is resistant to carbenicillin, the *tatA* (Figure 5A), *tatB* (Figure 5B), and *tatC* (Figure 5C) mutants were sensitive to the antibiotic. The introduction of plasmids containing a WT copy of *tatA* (*i.e.* pRB.TatA, Figure 5A) and *tatB* (*i.e.* pRB.TatB, Figure 5B) did not restore the ability of the *tatA* and *tatB* mutants to grow in the presence of carbenicillin, respectively. Resistance to the β-lactam was observed only when the *tatA* and *tatB* mutants were complemented with the plasmid specifying the entire *tatABC* locus (see pRB.TAT in Figure 5A and B), which is consistent with the results of the growth experiments presented in Figure 3. Introduction of the plasmid encoding only the WT copy of *tatC* (*i.e.* pRB.TatC) in the strain O35E.TC was sufficient to restore the growth of this *tatC* mutant on medium supplemented with carbenicillin (Figure 5C). Of note, the *tatC* mutant of strain O12E was tested in this manner and the results were consistent with those obtained with O35E.TC (data not shown). In order to provide an appropriate control for these experiments, an isogenic mutant strain of *M. catarrhalis* O35E was constructed in which the *bro-2* gene was disrupted with a kan^R^ marker. The mutant, which was designated O35E.Bro, grew at the same rate as the parent strain O35E in liquid medium (Figure 3C). As expected, the *bro-2* mutant did not grow on agar plates containing carbenicillin (Figure 5C).

**Figure 6:**
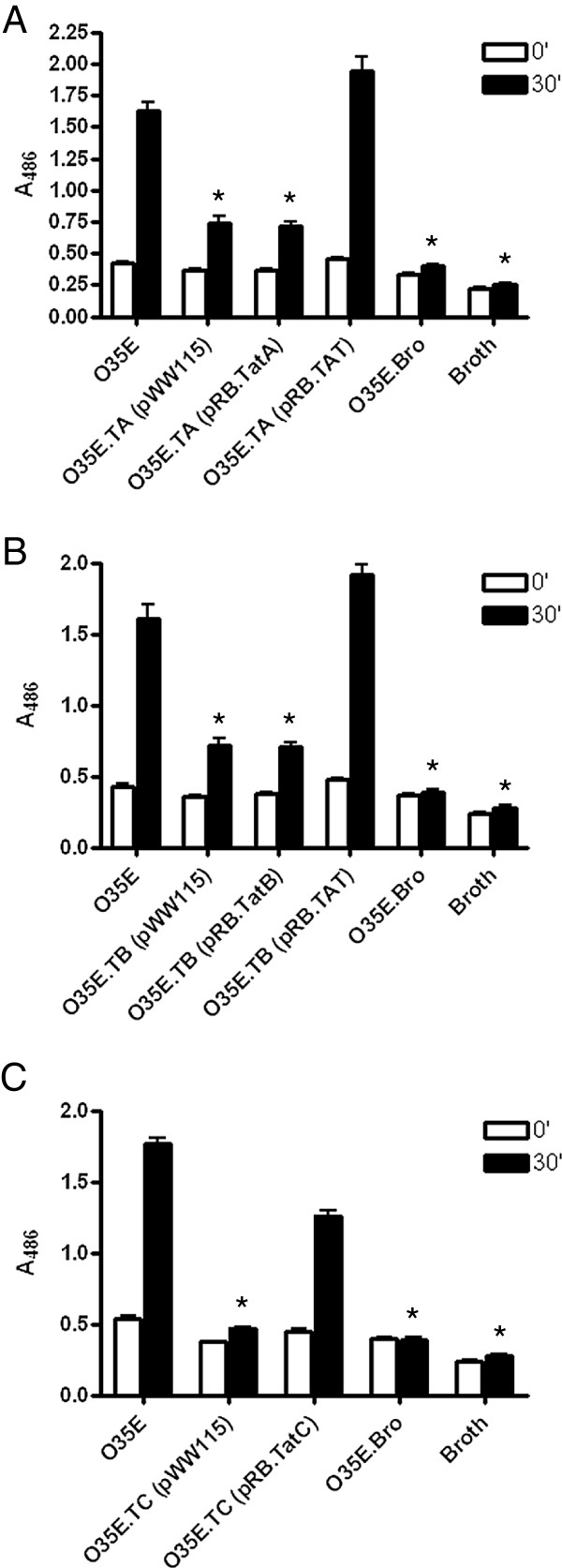
**Quantitative measurement of the β-lactamase activity produced by the *M. catarrhalis *WT isolate O35E and *tat *mutant strains.** The β-lactamase activity of strains was measured using the chromogenic compound nitrocefin. Bacterial suspensions were mixed with a 250 μg/mL nitrocefin solution and the absorbance at 486 nm (A_486_) was immediately measured and recorded as time “0” (open bars). The A_486_ of the samples was measured again after a 30-min incubation at room temperature (black bars). **Panel A**: The β-lactamase activity of O35E is compared to that of the *tatA* mutant strain, O35E.TA, carrying the plasmid pWW115 (control), pRB.TatA (specifies a WT copy of *tatA*), and pRB.TAT (harbors the entire *tatABC* locus). **Panel B**: The β-lactamase activity of O35E is compared to that of the *tatB* mutant, O35E.TB, carrying the plasmid pWW115, pRB.TatB (specifies a WT copy of *tatB*), and pRB.TAT. **Panel C**: The β-lactamase activity of O35E is compared to that of the *tatC* mutant, O35E.TC, carrying the plasmid pWW115 and pRB.TatC (contains a WT copy of *tatC*). The strain O35E.Bro, which lacks expression of the β-lactamase BRO-2, was used as a negative control in all experiments in addition to the broth-only control. The results are expressed as the mean A_486_ ± standard error. Asterisks indicate that the reduction in the β-lactamase activity of mutants is statistically significant (P < 0.05) when compared to the WT strain O35E.

The effect of *tat* mutations on the β-lactamase activity of *M. catarrhalis* was quantitatively measured using the chromogenic β-lactamase substrate nitrocefin. These assays were performed using suspensions of freshly plate-grown bacteria placed into the wells of a 48-well tissue culture plate. A solution containing nitrocefin was added to these suspensions and the change of color from yellow to red (indicative of cleavage of the β-lactam ring) was monitored by measuring the absorbance of well contents at a wavelength of 486 nm. Substantially less β-lactamase activity was observed for the *tatA, tatB* and *tatC* mutants compared to the WT strain O35E (Figure 6). Complementation of the *tatA* and *tatB* mutants with plasmids containing only the WT copies of the inactivated genes did not restore β-lactamase activity, as expected based on the results of the experiments depicted in Figures 3 and 5. The plasmid pRB.TAT, which specifies the entire *tatABC* locus, restored the ability of the mutants O35E.TA (Figure 6A) and O35E.TB (Figure 6B) to hydrolyze nitrocefin. The plasmid pRB.TatC was sufficient to rescue β-lactamase activity in the *tatC* mutant strain O35E.TC to near WT levels (Figure 6C). The *tatC* mutant of strain O12E was tested in this manner and the results were consistent with those obtained with O35E.TC (data not shown). The control strain, O35E.Bro, was impaired in its ability to hydrolyze nitrocefin at levels comparable to those of the *tatA*, *tatB* and *tatC* mutants (Figure 6A, B and C). Taken together, these results suggest that the *M. catarrhalis tatABC* locus is necessary for secretion of the β-lactamase BRO-2 into the periplasm where the enzyme can protect the peptidoglycan cell wall from the antimicrobial activity of β-lactam antibiotics.

**Figure 7:**
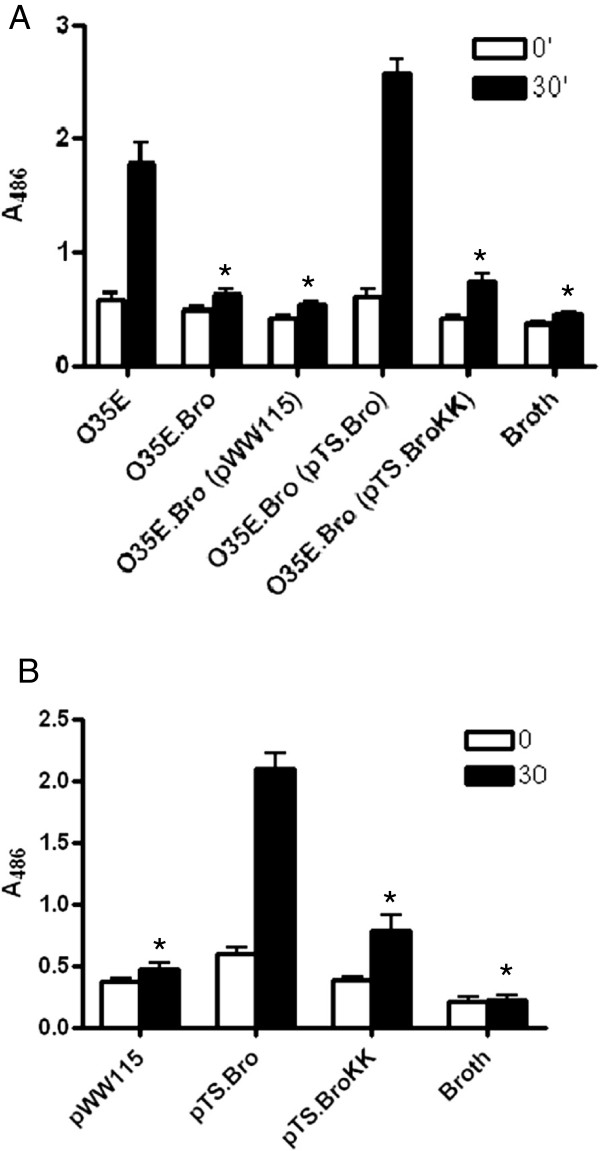
**Quantitative measurement of the β-lactamase activity of *M. catarrhalis *and recombinant *H. influenzae *strains.** β-lactamase activity was measured using the chromogenic compound nitrocefin. Bacterial suspensions were mixed with a 250 μg/mL nitrocefin solution and the A_486_ was immediately measured and recorded as time “0” (open bars). The A_486_ of the samples was measured again after a 30-min incubation at room temperature (black bars). **Panel A**: The β-lactamase activity of *M. catarrhalis* O35E is compared to that of the *bro-2* mutant, O35E.Bro, carrying plasmids pWW115, pTS.Bro, and pTS.BroKK. **Panel B**: The β-lactamase activity of *H. influenzae* DB117 carrying plasmids pWW115, pTS.Bro, and pTS.BroKK is compared. Sterile broth was used as a negative control in these experiments. The results are expressed as the mean ± standard error A_486_. Asterisks indicate that the reduction in the β-lactamase activity of strains lacking expression of BRO-2, or expressing a mutated BRO-2 that contains two lysine residues in its signal sequence instead of 2 arginines, is statistically significant (P < 0.05) when compared to bacteria expressing a WT copy of the *bro-2* gene.

To conclusively demonstrate that *M. catarrhalis* BRO-2 is secreted by the TAT system, we cloned the *bro-2* gene of strain O35E in the plasmid pWW115 (pTS.Bro) and used site-directed mutagenesis to replace the twin-arginine (RR) residues in BRO-2’s predicted signal sequence (Figure 4A) with twin lysine (KK) residues (pTS.BroKK). Similar conservative substitutions have been engineered in TAT substrates of other bacteria to demonstrate the importance of the RR motif in TAT-dependent secretion [74]. These plasmids were introduced in the mutant O35E.Bro and the recombinant strains were tested for their ability to hydrolyze nitrocefin. As shown in Figure 7A, expression of the mutated BRO-2 from plasmid pTS.BroKK did not restore the ability to hydrolyze nitrocefin. These results establish that the *M. catarrhalis* β-lactamase BRO-2 is secreted into the periplasm by the TAT system. Interestingly, the mutation in the RR motif of BRO-2 also interfered with secretion of the β-lactamase by recombinant *Haemophilus influenzae* DB117 bacteria (Figure 7B).

### Identification of other *M. catarrhalis* gene products potentially secreted by the TAT system

To identify other *M. catarrhalis* molecules that may be secreted via the TAT apparatus, the annotated genomic sequences of strains ATCC43617 [81] and BBH18 [78] were analyzed with prediction algorithms available through the TatFind 1.4 [82] and TatP 1.0 [83] servers. Although these servers are designed for the same purpose (*i.e.* identify proteins secreted by the TAT system), the algorithms used for each differ and as such proteins identified as TAT substrates do not overlap 100% between the two prediction algorithms.

**Figure 8:**
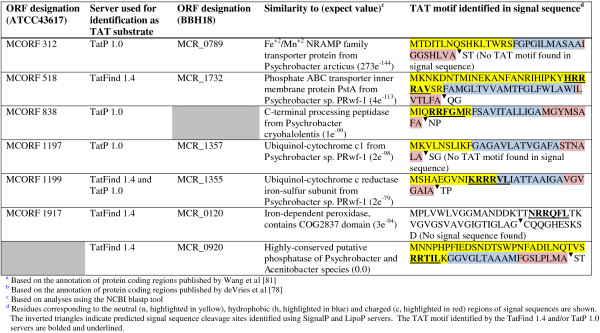
**Comparison of the putative TAT substrates identified in the genomes of *M. catarrhalis *strains ATCC43617**^**a **^**and BBH18**^**b**^**.**

Six ORFs were predicted to be TAT substrates in strain ATCC43617, only one of which was identified by both algorithms (Figure 8). The TatP 1.0 server identified MCORF 312 and MCORF 1197 as proteins potentially secreted by the TAT system, but no twin-arginine motif was found within the signal sequences of these gene products. Conversely, the TatFind 1.4 server identified MCORF 1917 as a TAT substrate and a twin-arginine motif was observed between residues 18 and 23. Although the encoded protein does not specify characteristics of a prokaryotic signal sequence (*i.e.* n-, h-, c-region), a potential lipoprotein signal sequence cleavage site was identified using the LipoP server. Interestingly, the MCORF 1197 and MCORF 1199 gene products resemble cytochrome c molecules involved in the electron transport chain. Cytochromes have been predicted, as well as demonstrated, to be TAT substrates in several bacterial species [84-87]. MCORF 1917 exhibits similarities to iron-dependent peroxidases, which is consistent with the previously reported role of the TAT system in the secretion of enzymes that bind metal ions, while MCORF 518 resembles the phosphate ABC transporter inner membrane protein PstA [88]. MCORF 838 shows similarities to a family of C-terminal processing peptidases and contains important functional domains including a post-translational processing, maturation and degradation region (PDZ-CTP), and a periplasmic protease Prc domain described as important for cell envelope biogenesis.

Six putative TAT substrates were identified in the genome of *M. catarrhalis* strain BBH18, five of which overlapping those predicted in ATCC43617 (Figure 8). Strain BBH18 specifies the unique TAT substrate MCR_920, which is predicted to be a highly-conserved phosphatase (Figure 8). The MCORF 1659 of strain ATCC43617 encodes a gene product that is 96.8% identical to this putative phosphatase, but neither of the TatFind 1.4 and TatP 1.0 servers identified the ORF as a TAT substrate, likely due to significant amino acid divergence in the signal sequence (data not shown). Strain BBH18 specifies a putative C-terminal processing peptidase (MCR_1063) that is 98.1% identical to the putative TAT substrate MCORF 838 of ATCC43617. Like the MCORF 838 of ATCC43617, the BBH18 gene product lacked a TAT motif in its signal sequence (data not shown). Despite these similarities, the BBH18 MCR_1063 C-terminal processing peptidase was not identified as a being potential TAT substrate by the TatFind 1.4 or TatP 1.0 algorithms.

## Conclusions

This report is the first characterization of a secretory apparatus for *M. catarrhalis*. Our data demonstrate that the TAT system mediates secretion of β-lactamase and is necessary for optimal growth of the bacterium. *Moraxella catarrhalis* is a leading cause of otitis media worldwide along with *Streptococcus pneumoniae* and non-typeable *Haemophilus influenzae* (NTHi), and is often found in mixed infections with these organisms [1-8,89]. In contrast to *M. catarrhalis*, most *S. pneumoniae* and NTHi isolates are susceptible to β-lactam antibiotics [90]. In a set of elegant studies, Schaar *et al.* demonstrated that outer membrane vesicles produced by *M. catarrhalis* contain β-lactamase and function as a long-distance delivery system to confer antimicrobial resistance for β-lactamase negative isolates of *S. pneumoniae* and NTHi [91]. This constitutes a novel mechanism by which *M. catarrhalis* promotes survival and infection by other pathogens in the context of polymicrobial disease. Hence, a greater understanding of the TAT secretion system of *M. catarrhalis* is a key area of future study as it may lead to the development of innovative strategies to improve the efficacy of existing antimicrobials used to treat bacterial infections by common childhood pathogens. Small molecular weight compounds that selectively inhibit TAT secretion in *M. catarrhalis* could be used in concert with β-lactam antibiotics as β-lactamase inhibitors. This hypothesis is supported by the recent discovery that the compounds *N*-phenyl maleimide and Bay 11–7782 specifically interfere with TAT-dependent secretion of the *Pseudomonas aeruginosa* phospholipase C PlcH [92].

## Methods

### Bacterial strains, plasmids, and growth conditions

Strains and plasmids are described in Table 1. *M. catarrhalis* was cultured using Todd-Hewitt (TH) medium (BD Diagnostic Systems) supplemented with 20 μg/mL kanamycin, 15 μg/mL spectinomycin, and/or 5 μg/mL carbenicillin, where appropriate. *Escherichia coli* was grown using Luria-Bertani (LB) medium (Fisher BioReagents) supplemented with 15 μg/mL chloramphenicol and/or 50 μg/mL kanamycin, where indicated. *Haemophilus influenzae* was cultured using Brain Heart Infusion (BHI) medium (BD Diagnostic Systems) supplemented with 50 mg/L hemin chloride (Sigma-Aldrich®) and 10 mg/L NAD (Sigma-Aldrich®) (BHI + Heme + NAD). This medium was further supplemented with 50 μg/mL spectinomycin where appropriate. Electrocompetent *M. catarrhalis* and *H. influenzae* cells were prepared as previously described [93]. All strains were cultured at 37°C in the presence of 7.5% CO_2_.

**Table 1:** Strains and plasmids used in this study

**Strain**	**Description**	**Source**
*M. catarrhalis*		
O35E	WT isolate from middle ear effusion (Dallas, TX)	[94]
O35E.TA	*tatA* isogenic mutant of strain O35E, kan^R^	This study
O35E.TB	*tatB* isogenic mutant of strain O35E, kan^R^	This study
O35E.TC	*tatC* isogenic mutant of strain O35E, kan^R^	This study
O35E.Bro	*bro-2* isogenic mutant of strain O35E, kan^R^	This study
O12E	WT isolate from middle ear effusion (Dallas, TX)	[28]
O12E.TC	*tatC* isogenic mutant of strain O12E, kan^R^	This study
McGHS1	WT isolate from patient with respiratory infection (Toledo, OH)	[33]
TTA37	WT isolate from transtracheal aspirate (Johnson City, TN)	[28]
V1171	WT isolate from nasopharynx of a healthy child (Chapel Hill, NC)	[28]
*H. influenzae*		
DB117	Host strain for cloning experiments with pWW115	[95,96]
*E. coli*		
EPI300	Cloning strain	Epicentre®
		Illumina®
Plasmids		
pCC1	Cloning vector, cam^R^	Epicentre®
		Illumina®
pCC1.3	pCC1-based plasmid containing kan^R^ marker, cam^R^kan^R^	[31]
pRB.TatA.5	Contains 886-nt insert specifying O35E *tatA* in pCC1, cam^R^	This study
pRB.TatB.1	Contains 858-nt insert specifying O35E *tatB* in pCC1, cam^R^	This study
pRB.TatC.2	Contains 1,018-nt insert specifying O12E *tatC* in pCC1, cam^R^	This study
pRB.TatC:kan	pRB.TatC.2 in which a kan^R^ marker disrupts the *tatC* ORF, cam^R^ kan^R^	This study
pRB.Tat.1	Contains 2,083-nt insert specifying O35E *tatABC* locus in pCC1, cam^R^	This study
pRB.TatA:kan	pRB.Tat.1 in which a kan^R^ marker disrupts the *tatA* ORF, cam^R^ kan^R^	This study
pRB.TatB:kan	pRB.Tat.1 in which a kan^R^ marker disrupts the *tatB* ORF, cam^R^ kan^R^	This study
pRN.Bro11	Contains 994-nt insert specifying O35E *bro-2* in pCC1, cam^R^	This study
pRB.Bro:kan	pRN.Bro11 in which a kan^R^ marker disrupts the *bro-2* ORF, cam^R^ kan^R^	This study
pTS.BroKK.Ec	pRN.Bro11 in which 2 arginines in the signal sequence of the *bro-2* gene product were replaced with 2 lysines by site-directed mutagenesis, cam^R^	This study
pWW115	*M. catarrhalis-H. influenzae* shuttle cloning vector, spc^R^	[95]
pRB.TatA	pWW115 into which the *tatA* insert of pRB.TatA.5 was subcloned, spc^R^	This study
pRB.TatB	pWW115 into which the *tatB* insert of pRB.TatB.1 was subcloned, spc^R^	This study
pRB.TatC	pWW115 into which the *tatB* insert of pRB.TatC.2 was subcloned, spc^R^	This study
pRB.TAT	pWW115 into which the *tatABC* locus of pRB.Tat.1 was subcloned, spc^R^	This study
pTS.Bro	pWW115 into which the *bro-2* insert of pRN.Bro11 was subcloned, spc^R^	This study
pTS.BroKK	pWW115 into which the *bro-2* insert of pTS.BroKK.Ec was subcloned, spc^R^	This study

### Recombinant DNA techniques

Standard molecular biology techniques were performed as described elsewhere [97]. *Moraxella catarrhalis* genomic DNA was obtained using the Easy-DNA™ kit (Invitrogen™ Life Technologies™) per the manufacturer’s instructions. Plasmid DNA was purified with the QIAprep Spin Miniprep system (QIAGEN).

Polymerase chain reactions were performed using *Taq* DNA Polymerase (Invitrogen™ Life Technologies™) unless otherwise specified. A 1,018-nt fragment containing the *tatC* gene was amplified with primers P1 (5′- AAAGCCAAGCCAACGGACTT-3′) and P2 (5′-ACCTCCAAGAAACCCACGCTATCA-3′) using genomic DNA from *M. catarrhalis* strain O12E (see Figure 1 for more details regarding primers). This PCR product was cloned into the vector pCC1 using the Copy Control™ PCR Cloning Kit (Epicentre® Illumina®) per the manufacturer’s instructions. This process yielded plasmid pRB.TatC.2, which was sequenced to verify that mutations were not introduced in the *tatC* gene during cloning. PCR products comprising *tatA* (886-nt in length), *tatB* (858-nt in length) and the entire *tatABC* locus (2,083-nt in length) were amplified with primers P3 (5′-AGGGCAACTGGCAAATTACCAACC-3′) and P4 (5′-AAACATGCCATACCATCGCCCAAG-3′), P5 (5′-CAAAGACTTGGGCAGTGCGGTAAA-3′) and P6 (5′-ATTCATTGGGCAGTAGAGCGACCA-3), and P7 (5′-CATCATTGCGGCCAAAGAGCTTGA-3′) and P8 (5′-AGCTTGCCGATCCAAACAGCTTTC-3′), respectively, using genomic DNA from *M. catarrhalis* strain O35E (see Figure 1 for more details regarding primers). These amplicons were cloned in the vector pCC1 as described above, producing plasmids pRB.TatA.5, pRB.TatB.1, and pRB.Tat.1. These constructs were sequenced to verify that mutations were not introduced in the *tat* genes during PCR. To examine conservation of the TatABC gene products, genomic DNA from *M. catarrhalis* strains O35E, O12E, McGHS1, V1171, and TTA37 was used to amplify 2.1-kb DNA fragments containing the entire *tatABC* locus with primer P7 and P8. These amplicons were sequenced in their entirety and the sequences were deposited in GenBank under accession numbers HQ906880 (O35E), HQ906881 (O12E), HQ906882 (McGHS1), HQ906883 (V1171), and HQ906884 (TTA37).

The *bro-2* gene specifying the β-lactamase of *M. catarrhalis* strain O35E was amplified with primers P9 (5′-TAATGATGCAACGCCGTCAT-3′) and P10 (5′-GCTTGTTGGGTCATAAATTTCC-3′) using Platinum® *Pfx* DNA Polymerase (Invitrogen™ Life Technologies™). This 994-nt PCR product was cloned into pCC1 as described above, generating the construct pRN.Bro11. Upon sequencing, the *bro-2* gene contained by pRN.Bro11 was found to be free of mutation. The nucleotide sequence of O35E *bro-2* was deposited in GenBank under the accession number JF279451.

### Mutant construction

To create a *tatC* mutation in *M. catarrhalis*, the plasmid pRB.TatC.2 was mutagenized with the EZ-TN5™ < KAN-2 > Insertion Kit (Epicentre® Illumina®) and introduced into Transformax™ EPI300™ electrocompetent cells. Chloramphenicol resistant (cam^R^, specified by the vector pCC1) and kanamycin resistant (kan^R^, specified by the EZ-TN5 < KAN-2 > TN) colonies were selected and plasmids were analyzed by PCR using the pCC1-specific primer, P11 (5′-TACGCCAAGCTATTTAGGTGAGA-3′), and primers specific for the kan^R^ marker, P12 (5′-ACCTACAACAAAGCTCTCATCAACC-3′) and P13 (5′-GCAATGTAACATCAGAGATTTTGAG-3′). This strategy identified plasmid pRB.TatC:kan, in which the EZ-TN5 < KAN-2 > TN was inserted near the middle of the *tatC* ORF. The disrupted *tatC* gene was then amplified from pRB.TatC:kan with the pCC1-specific primers P11 and P14 (5′-TAATACGACTCACTATAGGG-3′) using Platinum® *Pfx* DNA Polymerase. This 2.3-kb PCR product was purified and electroporated into *M. catarrhalis* strains O12E and O35E to create the kan^R^ isogenic mutant strains O12E.TC and O35E.TC via homologous recombination. Allelic replacement was confirmed by PCR with the *tatC* primers P1 and P2 using Platinum® *Pfx* DNA Polymerase. These primers yielded PCR products in the mutant strains that were 1.2-kb larger than the amplicons obtained in the wild-type (WT) isolates O35E and O12E due to the presence of the EZ-TN5 < KAN-2 > TN in *tatC*.

To construct mutations in the *tatA* and *tatB* genes of *M. catarrhalis* O35E, the plasmid pRB.Tat.1 was first mutagenized with the EZ-TN5™ In-Frame Linker Insertion Kit (Epicentre® Illumina®) and introduced into Transformax™ EPI300™ electrocompetent cells. Plasmid DNA was isolated from several cam^R^ (specified by the vector pCC1) and kan^R^ (specified by the EZ-TN5 < *Not* I/KAN-3 > TN) clones and sequenced to determine the sites of insertion of the TN. This approach identified the plasmids pRB.TatA:kan and pRB.TatB:kan, which contained the EZ-TN5 < *Not* I/KAN-3 > TN at nt 90 of the *tatA* ORF and nt 285 of the *tatB* ORF, respectively. These plasmids were then introduced into *M. catarrhalis* strain O35E by natural transformation as previously described [34]. The resulting kan^R^ strains were screened by PCR using primers specific for *tatA* (P3 and P4) and *tatB* (P5 and P6), which produced DNA fragments that were 1.2-kb larger in size in mutant strains when compared to the WT strain O35E because of the insertion of the EZ-TN5 < *Not* I/KAN-3 > TN in *tatA* and *tatB*. This strategy yielded the mutant strains O35E.TA and O35E.TB.

To construct a mutation in the *bro-2* gene of *M. catarrhalis* O35E, plasmid pRN.Bro11 was mutagenized with the EZ-TN5™ In-Frame Linker Insertion Kit as described above. Plasmids were isolated from kan^R^ cam^R^ colonies and sequenced to identify constructs containing the EZ-TN5 < *Not* I/KAN-3 > TN near the middle of the *bro-2* ORF. This approach yielded the construct pRB.Bro:kan, which was introduced in *M. catarrhalis* O35E by natural transformation. Transformants were selected for resistance to kanamycin and then tested for their ability to grow on agar plates containing the β-lactam antibiotic carbenicillin. Kan^R^ and carbenicillin sensitive (cab^S^) strains were further analyzed by PCR using primers P9 and P10 to verify allelic exchange of the *bro-2* gene. These primers produced a 1-kb DNA fragment in the WT strain O35E and a 2.2-kb in the mutant O35E.Bro, which is consistent with insertion of the 1.2-kb EZ-TN5 < *Not* I/KAN-3 > TN in *bro-2*.

### Site-directed mutagenesis of the *M. catarrhalis bro-2* gene

The *bro-2* ORF of *M. catarrhalis* O35E harbored by plasmid pRN.Bro11 was mutated using the QuikChange Lightning Site-Directed Mutagenesis Kit (Agilent Technologies) according to the manufacturer’s instructions. The mutagenesis primers, P15 (5′- AAGGGGATAATGATGCAAAAGAAGCATTTTTTA-3′) and P16 (5′-GGTTTTTTGTAAAAAATGCTTCTTTTGCAT CAT-3′), were used to replace two arginine residues at position 4 and 5 of BRO-2 with two lysines, yielding plasmid pTS.BroKK.Ec. This plasmid was sequenced to verify that only the intended mutations were introduced in the *bro-2* ORF.

### Complementation of mutants

The construction of plasmids to complement *tat* and *bro2* mutant strains was achieved as follows. Plasmid DNA (pRB.TatA.5, pRB.TatB.1, pRB.TatC.2, pRB.Tat.1, pRN.Bro11, pTS.BroKK.Ec) was digested with *BamHI* to release the cloned *M. catarrhalis* genes from the vector pCC1. Gene fragments were purified from agarose gel slices using the High Pure PCR Product Purification Kit (Roche Applied Science), ligated into the *BamHI* site of the *M. catarrhalis*/*Haemophilus influenza*-compatible shuttle vector pWW115 [95], and electroporated into *H. influenzae* strain DB117. Spectinomycin resistant (spc^R^) colonies were screened by PCR using the pWW115-specific primers P17 (5′-TACGCCCTTTTATACTGTAG-3′) and P18 (5′-AACGACAGGAGCACGATCAT-3′), which flank the *BamHI* cloning site, to identify clones containing inserts of the appropriate size for the *tat* and *bro2* genes. This process produced plasmids pRB.TatA, pRB.TatB, pRB.TatC, pRB.TAT, pTS.Bro, and pTS.BroKK. The O35E.TA mutant was naturally transformed with plasmids pWW115, pRB.TatA, and pRB.TatABC. The plasmids pWW115, pRB.TatB, and pRB.TAT were introduced in the O35E.TB mutant by natural transformation. The *tatC* mutants O35E.TC and O12E.TC were naturally transformed with the vector pWW115 and plasmid pRB.TatC. The plasmids pWW115, pTS.Bro, and pTS.BroKK were electroporated into the *bro-2* mutant strain O35E.Bro. The successful introduction of these plasmids into the indicated strains was verified by PCR analysis of spc^R^ transformants with the pWW115-specific primers P17 and P18, and by restriction endonuclease analysis of plasmid DNA purified from each strain.

### Growth rate experiments

*Moraxella. catarrhalis* strains were first cultured onto agar plates supplemented with appropriate antibiotics. These plate-grown bacteria were used to inoculate 500-mL sidearm flasks containing 20-mL of broth (without antibiotics) to an optical density (OD) of 50 Klett units. The cultures were then incubated with shaking (225-rpm) at a temperature of 37°C for 7-hr. The OD of each culture was determined every 60-min using a Klett™ Colorimeter (Scienceware®). These experiments were repeated on at least three separate occasions for each strain. In some experiments, aliquots were taken out of each culture after recording the optical density, diluted, and spread onto agar plates to determine the number of viable colony forming units (CFU).

### Carbenicillin sensitivity assays

*Moraxella catarrhalis* strains were first cultured onto agar plates supplemented with the appropriate antibiotics. These plate-grown bacteria were used to inoculate sterile Klett tubes containing five-mL of broth (without antibiotics) to an OD of 40 Klett units. Portions of these suspensions (25 μL) were spotted onto agar medium without antibiotics as well on plates supplemented with carbenicillin, and incubated at 37°C for 48-hr to evaluate growth. Each strain was tested in this manner a minimum of three times.

### Nitrocefin assays

*Moraxella catarrhalis* and *H. influenzae* strains were tested for their ability to cleave the chromogenic β-lactamase substrate nitrocefin as previously described [98]. Bacterial strains were first cultured onto agar plates supplemented with appropriate antibiotics. These plate-grown cells were suspended to an OD of 300 Klett units in 5-mL of broth, and aliquots (50 μL, ~10^7^ CFU) were transferred to duplicate wells of a 48-well tissue culture plate; control wells were seeded with broth only. To each of these wells, 325 μL of a nitrocefin (Calbiochem®) solution (250 μg/mL in phosphate buffer) was added and the absorbance at a wavelength of 486 nm (A_486_) was immediately measured using a μQuant™ Microplate Spectrophotometer (BioTek®) and recorded as time “0”. The A_486_ of the samples was then measured after a 30-min incubation at room temperature. These experiments were repeated a minimum of three times for each strain.

### Sequence analyses and TAT prediction Programs

Sequencing results were analyzed and assembled using Sequencher® 4.9 (Gene Codes Corporation). Sequence analyses and comparisons were performed using the various tools available through the ExPASy Proteomics Server (http://au.expasy.org/) and NCBI (http://blast.ncbi.nlm.nih.gov). To identify potential TAT substrates of *M. catarrhalis*, annotated nucleotide sequences from strain ATCC43617 [81] were translated and analyzed with the prediction algorithms available through the TatFind 1.4 (http://signalfind.org/tatfind.html) [82] and TatP 1.0 (http://www.cbs.dtu.dk/services/TatP/) [83] servers using the default settings. The published genomic sequence of *M. catarrhalis* strain BBH18 [78] was analyzed in the same manner.

### Statistical analyses

The GraphPad Prism Software was used for all statistical analyses. Growth rate experiments and nitrocefin assays were analyzed by a two-way analysis of variants (ANOVA), followed by the Bonferroni post-test of the means of each time point. Asterisks indicate statistically significant differences where *P* < 0.05.

## Abbreviations

ORF: Open reading frame; kanR: Kanamycin-resistance; spcR: Spectinomycin-resistance; camR: Chloramphenicol-resistance; cabR: Carbenicillin-resistance; cabS: Carbenicillin-sensitive; A486: Absorbance at a wavelength of 486 nm, TAT, twin-arginine translocation; MW: Molecular weight; CFU: Colony forming units; OD: Optical density; aa: Amino acid.

## Competing interests

RB, TLS and ERL do not have financial or non-financial competing interests. In the past five years, the authors have not received reimbursements, fees, funding, or salary from an organization that may in any way gain or lose financially from the publication of this manuscript, either now or in the future. Such an organization is not financing this manuscript. The authors do not hold stocks or shares in an organization that may in any way gain or lose financially from the publication of this manuscript, either now or in the future. The authors do not hold and are not currently applying for any patents relating to the content of the manuscript. The authors have not received reimbursements, fees, funding, or salary from an organization that holds or has applied for patents relating to the content of the manuscript. The authors do not have non-financial competing interests (political, personal, religious, ideological, academic, intellectual, commercial or any other) to declare in relation to this manuscript.

## Authors’ contributions

RB helped conceive the study, participated in its design and coordination, performed most of the experiments, and helped with redaction of the manuscript. TLS performed the experiments pertaining to site-directed mutagenesis of *bro-2* and functional analysis of the mutated gene product. ERL conceived the study, participated in its design and coordination, and helped with redaction of the manuscript. All authors read and approved the final manuscript.
